# Harnessing Natural Killer Cells: From Neuroimmunology to Novel Therapies for Central Nervous System Diseases

**DOI:** 10.1002/mco2.70983

**Published:** 2026-09-21

**Authors:** Kaihang Deng, Wai Po Chong

**Affiliations:** ^1^ School of Chinese Medicine Hong Kong Baptist University Hong Kong China; ^2^ Institute for Research and Continuing Education Hong Kong Baptist University Shenzhen China

**Keywords:** adoptive immunotherapy, central nervous system, glioblastoma, natural killer cells, neuroinflammatory diseases

## Abstract

Natural killer (NK) cells are innate lymphocytes that defend the host by directly killing virus‐infected, stressed, and tumor cells. Their direct cytotoxic mechanisms include the release of cytotoxic granules, engagement of death receptor pathways, and mediation of antibody‐dependent cellular cytotoxicity (ADCC). They can also eliminate targets indirectly via the secretion of proinflammatory cytokines. Importantly, recent studies show NK cells help limit autoimmunity by targeting autoreactive immune cells, highlighting their broader role in maintaining tissue homeostasis, including within the immune‐privileged central nervous system (CNS). Both preclinical and clinical studies support the therapeutic potential of NK cell‐based immunotherapies for neuroinflammatory, neurodegenerative, and neuro‐oncologic diseases. A key challenge remains achieving sufficient specificity and efficacy within the CNS, which necessitates a more profound understanding of NK cell biology in the unique brain microenvironment. This review summarizes the fundamental biology of NK cells and their roles in CNS pathologies, with a focus on their interactions with the CNS niche. We also discuss recent advances, such as immune‐engager strategies and adoptive NK cell therapies, aiming to provide insights to guide future therapeutic development for CNS disorders.

## Graphical abstract

This review delineates the multifaceted roles of natural killer (NK) cells in the central nervous system, spanning direct cytolysis, immunoregulation, and crosstalk with the brain microenvironment. We evaluate the translational potential and challenges of NK cell immunotherapies for a spectrum of neurological conditions.

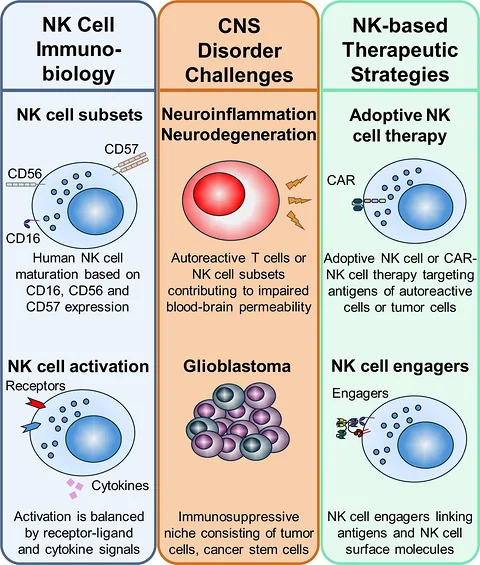

## 1 Introduction

Natural killer (NK) cells, discovered in the early 1970s as the first innate lymphoid cells (ILCs), are defined as CD3^−^CD56^+^ in humans and CD3^−^NKp46^+^ in mice. Initially identified by their ability to kill tumor cells without prior sensitization, these innate lymphocytes remain indispensable players in the ILC family [1, 2, 3]. Though all ILCs produce cytokines for immunoregulation, NK cells additionally exhibit cytolytic activities through the expression of granzymes and perforin, which have been suggested as the counterparts of CD8^+^ T cells [4]. Nonetheless, unlike the CD8^+^ T cells activated with prior priming by VDJ rearrangement, NK cells recognize target cells and respond with activation or inhibition signals intrinsically based on the germline‐encoded surface receptors, including NKp46, NKG2D, CD16, CD94/NKG2A, killer cell immunoglobulin‐like receptors (KIRs), lymphocyte function‐associated antigen‐1 (LFA‐1), and others [5]. Based on the innate cytotoxicity to virus‐infected cells and stressed cells, NK cells are promising candidates for the development of off‐the‐shelf cell‐based immunotherapeutic products. Indeed, translational research has harnessed allogeneic NK cell infusion therapy and chimeric antigen receptor NK cell (CAR‐NK) therapy for hematologic malignancies and some solid tumors [6, 7, 8].

The therapeutic and biological success of NK cells in the periphery has intensely stimulated the investigation of their roles within the central nervous system (CNS), an exploration parallel to the ongoing paradigm shift in CNS “immune privilege.” For decades, the brain was viewed as an immunologically isolated site secured by the blood–brain barrier (BBB) and blood–cerebrospinal fluid barrier (BCSFB). Milestone discoveries have since revised this dogma, revealing that NK cells actively traffic through and reside within either homeostatic or neurological niches, such as the meninges and cerebrospinal fluid, to participate in neuroprotection and surveillance [9, 10, 11, 12]. Consequently, mapping NK cell operations within this regulated neuro‐immune network has emerged as a major frontier in translational neuroscience.

This neuro‐immune network exhibits profound microenvironmental specificity under pathological conditions, where the precise composition of the CNS immune infiltrate varies dramatically across diseases. In the context of neuroinflammation, autoreactive T cells and microglia are the predominant populations, whereas in a neuro‐oncogenesis background, myeloid‐derived suppressor cells (MDSCs) and tumor‐associated macrophages dominate the immune cell infiltrate, which are often prioritized in research due to the percentage and functional significance [13, 14, 15]. In contrast, though NK cells are relatively scarce, they contribute disproportionately to tissue fate. Uniquely poised by a rapid response, these effectors participate significantly in both CNS homeostasis and disease processes through their innate cytotoxic activity and versatile immunoregulatory functions.

The expanding body of foundational and clinical literature provides a solid baseline to characterize NK cell dynamics within the CNS. As individual studies and related reviews continue to enrich this field, a systematic synthesis focused specifically on the continuous life cycle of these effectors across diverse neuropathological contexts remains highly valuable. To provide a clear understanding of NK cell subsets in CNS disorders, this review transitions logically from basic biology to clinical translation.

We begin by outlining the phenotypic origin, maturation, and tissue‐resident development of NK cells. Next, we dissect the physiological crosstalk between NK cells and the CNS niche under normal brain homeostasis. We then evaluate the protective and detrimental roles of distinct NK cell subsets in neuroinflammatory diseases and neuro‐oncogenesis. Finally, we discuss current technical hurdles alongside emerging frontiers shaping the next phase of neuroimmunotherapy and drug development.

## 2 NK Cell Biology and Subsets

NK cell biology centers on a structured spectrum of developmental trajectories and phenotypic variations, dictating how distinct subsets mature, differentiate, and execute their specialized effector functions.

### 2.1 Ontogeny and Heterogeneity of Human NK Cells

In humans, NK cells originate from CD34^+^ hematopoietic progenitor cells in the bone marrow [16]. Driven by lineage‐specific transcription factors such as T‐bet and Eomes, these lymphoid progenitors downregulate CD34 and progress through sequential natural killer progenitor stages to yield mature CD34^−^CD3^−^CD56^+^ NK cells [17, 18, 19]. Subsequently, NK cells egress from bone marrow toward peripheral organs such as the lung, lymph nodes, spleen, and liver [20].

In the periphery, circulating human NK cells are divided into two classical functional subsets that reflect distinct maturation stages based on their surface density of CD16 and CD56 [21]. The cytotoxic (CD16^bright^CD56^dim^) NK cells account for 90% of peripheral NK cells with potent cytotoxicity due to the lytic granules prepackaged with perforin and granzymes as well as antibody‐dependent cellular cytotoxicity (ADCC) [22]. Conversely, the less mature CD16^dim^CD56^bright^ subsets constitute a small part in circulating NK cells and are primarily characterized by their ability to regulate immune responses by the production of numerous cytokines, including interferon‐gamma (IFN‐γ), tumor necrosis factor‐alpha (TNF‐α), granulocyte‐macrophage colony‐stimulating factor (GM‐CSF), interleukin‐10 (IL‐10), interleukin‐13 (IL‐13), granulocyte colony‐stimulating factor (G‐CSF), and macrophage colony‐stimulating factor (M‐CSF) [23]. During terminal differentiation, human CD56^dim^ NK cells progressively acquire CD57, a marker of functional senescence and high cytolytic capacity [24, 25]. Crucially, while CD57 expression denotes highly mature NK cells, it also marks a specialized population of adaptive or memory NK cells. Typically elicited by viral infections such as human cytomegalovirus, these adaptive NK cells are characterized by the co‐expression of CD57 and the activating receptor NKG2C, suggesting that CD57 serves as a key marker to identify “memory” NK cells that have expanded in response to infection [26].

### 2.2 Developmental Trajectories and Functional Homology in Mice

In mice, the maturation cascade of NK cells follows a distinct yet functionally parallel track. Murine progenitors sequentially undergo refined developmental stages characterized by the induction of CD122 expression, transitioning from immature NK cells (CD27^+^CD11b^−^, CD27^+^CD11b^+^) to mature NK cells (CD27^−^CD11b^+^) [27, 28, 29]. Parallel to human maturation, KLRG1 serves as the definitive marker for terminal differentiation in mouse NK cells, mirroring human CD57 dynamics [30, 31, 32].

Despite the divergence in superficial surface markers between species, human and murine NK cell subsets exhibit striking transcriptomic and functional alignment (Table 1). Specifically, the mouse CD11b^+^CD27^+^ subset corresponds closely to the human CD16^dim^CD56^bright^ phenotype in terms of cytokine production profiles, whereas the highly cytotoxic mouse CD11b^+^CD27^−^ subset aligns with the mature human CD16^bright^CD56^dim^ counterpart. Meanwhile, the CD11b^−^CD27^+^ population represents an immature intermediary within the murine maturation hierarchy [33].

**TABLE 1:** NK cell developmental stages in human and mouse.

Stage	Human surface receptor repertoire	Mouse surface receptor repertoire	References
Common lymphocyte progenitor	Lin^−^ CD34^+^ CD38^+^ CD244^+^ CD45RA^+^ CD127^+^	Lin^−^ CD34^+^ Sca‐1^low^ CD117^low^ Flt3^+^ CD127^+^	[18, 19, 27, 28]
NK progenitor	Lin^−^ CD38^+^ CD45RA^+^ CD127^+^ CD122^+^ NKG2D^+^ IL‐1R1^+^	Lin^−^ CD117^low^ CD127^+^ CD27^+^ CD244^+^ CD122^−^	[19, 34]
Refined NK progenitor	/	Lin^−^ CD127^+^ CD27^+^ CD244^+^ CD122^+^ NKG2D^+^	[27]
Immature NK cell	IL‐2Rβ^+^ NKG2D^+^ NKG2A^+^ IL‐1R1^+^ NKp30^+^ NKp46^+^ KLRB1^+^ KIR^−^ CD56^bright^ CD16^dim^	CD27^+^ CD244^+^ CD127^−^ NK1.1^+^ NKG2D^+^ NKp46^+^ CD11b^−^ Ly49^+^ CD49b^+^	[22, 29, 33]
Mature NK cell	IL‐2Rβ^+^ NKG2D^+^ IL‐1R1^+^ NKp30^+^ NKp46^+^ KLRB1^+^ KIR^−^ CD56^dim^ CD16^bright^	CD27^+^ CD244^+^ CD127^−^ NK1.1^+^ NKG2D^+^ NKp46^+^ CD11b^+^ Ly49^+^ CD49b^+^	[22, 29]
Terminally mature NK cell	IL‐2Rβ^+^ NKG2D^+^ IL‐1R1^+^ NKp30^+^ NKp46^+^ KLRB1^+^ KIR^+^ CD56^dim^ CD16^bright^ CD57^+^	CD27^−^ CD244^+^ CD127^−^ NK1.1^+^ NKG2D^+^ NKp46^+^ CD11b^+^ Ly49^+^ CD49b^+^ KLRG1^+^	[24, 25, 30]

Abbreviations: CD, cluster of differentiation; Flt3, FMS‐like tyrosine kinase 3; IL, interleukin; IL‐1R1, interleukin‐1 receptor type 1; IL‐2Rβ, interleukin‐2 receptor subunit beta; KIR, killer cell immunoglobulin‐like receptor; KLRB1, killer cell lectin‐like receptor subfamily B member 1; KLRG1, killer cell lectin‐like receptor G1; Lin, lineage markers; Ly49, lymphocyte antigen 49; NK1.1, natural killer cell surface antigen killer cell lectin‐like receptor subfamily B member 1C (KLRB1C); NKG2A/D, natural killer group 2 member A/D; NKp30/46, natural killer cell p30/46‐related protein; Sca‐1, stem cells antigen‐1.

Beyond the circulating NK cells, the diversity of NK cells extends to tissue‐resident NK (trNK) cells, such as trNK cells in the thymus, liver, skin, and uterus. Certain trNK cells may develop memory, like memory‐like NK cells. For instance, the mouse intrahepatic trNK cells (CD49a^+^DX5^−^) possessed memory potential and conferred antigen‐specific responses after challenge [35]. Skin trNK cells (Tcf1^hi^CD69^hi^) are derived from circulating NK cells triggered by viral and bacterial infections, localize permanently at sites of previous infection, and rapidly acquire effector function to respond to secondary infection [36]. CNS trNK cells reside in the cerebrospinal fluid (CSF) and increase to 50% of the NK population during monoclonal antibody (mAb) therapy, while cytotoxic NK cells, immature NK cells, and ILC subsets all decrease, suggesting that trNK cells undergo specialization in the CNS and are functionally distinct from the circulating NK cells, as detailed in Section 4.1 [37]. Overall, growing research on trNK cells further blurs the boundaries between ILC and NK cells, while also raising questions about their developmental origins and the signals necessary for maintaining tissue residency [38].

In summary, despite divergent surface receptors, human and murine NK cells share a functional spectrum from immature cytokine producers to mature cytotoxic effectors and memory subsets. This regulated tissue heterogeneity establishes the baseline for NK cell biology.

## 3 NK Cell Activation

Beyond subset lineage, the execution of their niche‐specific functions remains fundamentally governed by the integration of NK cell surface signaling. Crucially, mapping these subset responses requires a precise understanding of the core receptor–ligand balances that dictate NK cell activation.

**FIGURE 1:**
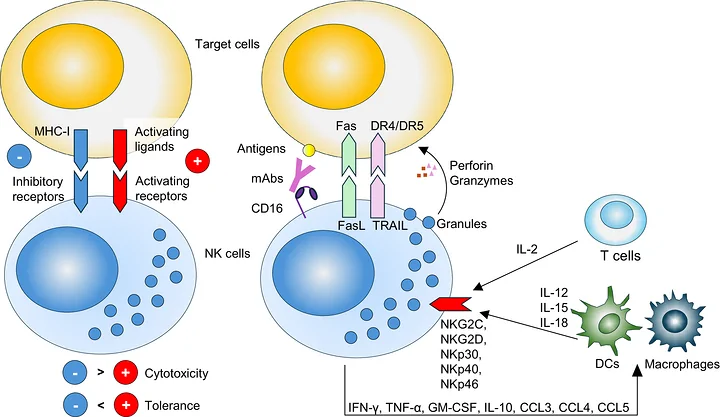
The activation and function effects of NK cells. A dynamic balance between activating and inhibiting receptors guarantees the control of NK cell effector activity. NK cell activation can happen when there are fewer MHC I molecules on target cells or when there are more activation ligands on those cells (left panel). NK cells can be further activated by cytokines secreted from other immune cells. In turn, NK cells secrete cytokines and chemokines to recruit other immune cells for coordination. There are three ways to induce apoptosis in target cells: Cell death caused by perforin and granzymes; Fas‐mediated and TRAIL‐mediated apoptosis; and CD16‐dependent ADCC (right panel). MHC‐I, major histocompatibility complex class I; mAbs, monoclonal antibodies; CD16, cluster of differentiation 16; FasL, Fas ligand; TRAIL, TNF‐related apoptosis‐inducing ligand; DR4/DR5, death receptor 4/death receptor 5; IL, interleukin; IFN‐γ, interferon‐gamma; TNF‐α, tumor necrosis factor‐alpha; GM‐CSF, granulocyte‐macrophage colony‐stimulating factor; CCL, C‐C motif chemokine ligand; NKG2C/D, natural killer group 2 member C/D; NKp30/40/46, natural killer cell p30/p40/p46‐related receptors; ADCC, antibody‐dependent cellular cytotoxicity.

The dynamic equilibrium between activation and inhibitory receptors on the cell membrane regulates NK cell activity. Activation of NK cells occurs through the engagement of activating receptors, including lymphocyte antigen 49D (Ly49D), lymphocyte antigen 49H (Ly49H) in mice, activating subtypes of KIRs in humans, NKG2D, NKp30, NKp44, and NKp46 in both species [39, 40, 41]. The inhibitory receptors, including Ly49A/C/I/P in mice, KIRs in humans, and the CD94/NKG2A heterodimer in both species, identify many variants of major histocompatibility complex class I (MHC‐I) molecules [42]. Consequently, a decline of MHC‐I molecules or upregulation of ligands for activating receptors on target cells will attenuate the inhibitory signal of NK cells, hence facilitating NK cell activation (Figure 1).

Cytokine supplements further enhance NK cell activation. NK cells maintain reactivity to interleukin‐2 (IL‐2) due to the constitutive expression of the IL‐2 receptor. IL‐2, mainly secreted by activated CD4^+^ T cells, can promote the survival, activation, and cytotoxicity of NK cells within a short period. Interleukin‐15 (IL‐15), secreted by dendritic cells (DCs), macrophages, and other myeloid cells, is a promising alternative to IL‐2, effectively stimulating NK cells without activating regulatory T cells (Tregs). Furthermore, the administration of IL‐15 alone can sustain the NK cells’ cytolytic function following infusion in patients with advanced cancers (NCT01572493) [43]. Both IL‐2 and IL‐15 induce the expression of NK cell activation receptors, including NKG2D, NKp30, NKp44, and NKp46. The combined use of IL‐2 and IL‐15 can enhance the proliferation and activity of NK cells, thereby increasing NK cell cytotoxicity toward tumor cells. IL‐12 involves the generation of memory or memory‐like NK cells by activation of signal transducer and activator of transcription 4 (STAT4) signals [44]. IL‐18 acts as a costimulatory cytokine that functions with IL‐12 and IL‐15 synergistically, and additionally enhances NK cell effector functions, including IFN‐γ secretion [45, 46, 47].

Once primed, NK cells secrete lytic granules containing perforin and granzymes to induce apoptosis in target cells. NK cells also express the Fas ligands (FasL) and tumor necrosis factor‐related apoptosis‐inducing ligands (TRAIL) to activate apoptosis signaling pathways through Fas and DR4 and DR5, respectively [48]. NK cells produce cytokines, e.g., IFN‐γ, TNF‐α, GM‐CSF, IL‐10, and chemokines, e.g., C–C motif chemokine ligand 3 (CCL3), CCL4, CCL5, to recruit DCs and macrophages for a coordinated immune response [49, 50, 51]. Besides, NK cells facilitate ADCC via Fc receptors CD16 and CD32, which initiate lytic activity on encountering mAb‐coated targets and trigger cytotoxicity and cytokine release (Figure 1) [52].

In short, NK cell activation is a finely tuned process balancing cell‐surface receptors, cytokine priming, and direct effector execution. While these foundational activation principles operate globally, their regulatory dynamics undergo profound spatial reorganization when cells enter specialized tissue compartments, particularly within the CNS.

## 4 NK Cells Dynamics in the CNS

NK cells actively orchestrate the CNS immune landscape through chemokine‐driven recruitment into specialized niches and intense crosstalk with the neural stroma. This microenvironmental integration ultimately drives a functional dualism, shifting NK cells between protective immunosurveillance and neurotoxic damage depending on the pathological context.

### 4.1 Recruitment Pathways and Anatomical Niches of CNS NK Cells

**FIGURE 2:**
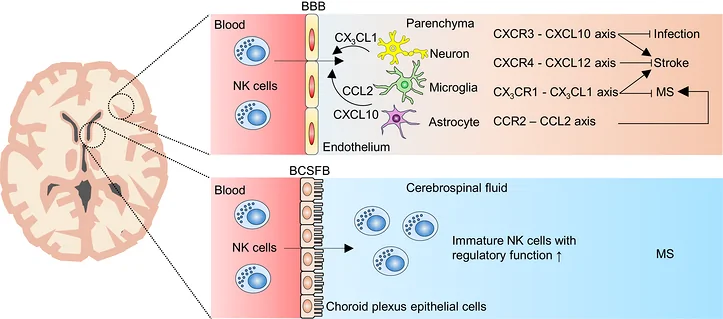
Trafficking routes of NK cells in the CNS by trespassing the BBB or BCSFB. Neurons, microglia, and astrocytes secrete chemokines such as CX3CL1, CXCL10, and CCL2 to induce NK cells to traffic into the parenchyma through the BBB. NK cells migrate into brain lesion sites and exert neuroprotective or proinflammatory effects under different chemotactic axes. The choroid plexus forms BCSFB, through which immature NK cells transmigrate via chemokine secretion to dominate in the CSF. BBB, blood–brain barrier; BCSFB, blood–cerebrospinal fluid barrier; CCL2, C‐C motif chemokine ligand 2; CCR2, C‐C motif chemokine receptor 2; CXCL10, C‐X‐C motif chemokine ligand 10; CXCL12, C‐X‐C motif chemokine ligand 12; CX3CL1, C‐X3‐C motif chemokine ligand 1; CXCR3, C‐X‐C motif chemokine receptor 3; CXCR4, C‐X‐C motif chemokine receptor 4; CX3CR1, C‐X3‐C motif chemokine receptor 1; MS, multiple sclerosis. Some graphical elements were adapted from Servier Medical Art (https://smart.servier.com/).

The BBB and BCSFB usually keep peripheral immune cells away from the CNS by forming tight junctions between blood vessel endothelial cells and choroid plexus (CP) epithelial cells [53]. However, peripheral macrophages and DCs can be located at the blood–brain interface and perform immune surveillance in the CNS by promoting antigen presentation to T cells. This process allows activated T cells to enter the CNS and eliminate harmful substances [54, 55, 56, 57, 58]. While NK cells are sparsely distributed in the CNS under homeostatic conditions, their recruitment and activation are significantly enhanced during pathological states such as infection or ischemia. It is plausible that NK cells may migrate into the CNS in a similar manner (Figure 2). For instance, C‐X‐C chemokine receptor 3 (CXCR3)‐positive NK cells accumulate in the ischemic brain in a C‐X‐C motif chemokine ligand 10 (CXCL10) dose‐dependent way and may accelerate BBB injury. On the other hand, NK cell accumulation in the CNS as well as increased survival were observed in RAG1^−/−^ mice inoculated with a CXCL10‐expressing coronavirus, suggesting the CXCL10‐CXCR3 axis plays a pivotal role in NK cell recruitment into the CNS to defend against coronavirus infection [56, 59, 60]. Another study demonstrated that the CXCL12–CXCR4 axis activation that happened in the BBB can attract NK cells to ischemic brain lesions, and infused NK cells protected the mouse brains and improved motor movement after stroke induction [61].

Beyond the acute, inflammation‐driven infiltration of these peripheral effectors, distinct cohorts of NK cells safely reside as specialized brain‐resident populations within the central nervous system borders under steady‐state conditions. Regarding spatial localization, single‐cell RNA sequencing has confirmed that NK cells consistently exist in the subdural meninges, dura mater, and CP [10]. Further characterizing these compartments, a landmark study by Korin B et al. demonstrated that resident NK cells are present at lower frequencies (1.1 ± 0.14%) in the naïve mouse meninges and CP, characterized by CD45^hi^CD49b^+^ expression. In terms of proportion, NK cells in the brains (2.78 ± 0.27%) are significantly fewer than the peripheral counterparts (4.57 ± 0.27%) among the CD45^high^ immune cells. Despite the low abundance, these CNS‐associated NK cells are heavily skewed toward a distinct, highly mature, and functional subset. Specifically, NK cells in the brain compartment exhibit an approximately sevenfold higher proportion of IL‐2R^+^CD27^+^ cells compared with peripheral blood (brain: 41.2 ± 2.82%; blood: 5.26 ± 0.99%). This CD27^high^ profile has been previously associated with increased effector function and elevated responsiveness to chemokines. This functional readiness is further supported by significantly elevated levels of CD62L on brain‐resident NK cells (50.05 ± 3.77%) compared with those in the circulation (23.25 ± 3.48%), a marker linked to a relatively mature NK cell phenotype and stronger cytotoxic capabilities [9]. Consistently, CX3CR1^+^ NK cell subsets are more abundant in the brain than in the blood, indicating a greater potential for activation by CX3CL1 and enhanced cytotoxicity against autoreactive T cells in the CNS, thereby exerting neuroprotective effects [9, 11, 62, 63]. In line with the murine brain data, human resident NK cells are present within the dura mater of meninges, the CP stroma, and the CSF at steady states [12, 64, 65]. Under physiological conditions, these cells display a resting CD45RA^high^ phenotype with high cytotoxic potential (CD56^dim^T‐bet^high^), but maintain low baseline abundance and poor migratory activity (CD54^low^CD31^low^) [64]. In sharp contrast to this quiescent steady state, donors with neurological diseases exhibit divergent NK subset dynamics. Intriguingly, the data from donors with neurological disease showed that CD56^dim^ NK cells are more prevalent in the CP than CD56^bright^ NK cells, but the reverse is seen in the CSF, confirming the distinct roles of NK cell subsets—cytotoxic CD56^dim^ NK cells resolve CNS damage, while CD56^bright^ NK cells engage in local immune surveillance (Figure 2) [12, 64, 66]. Beyond these classical boundaries, the immunological landscape of the CNS extends to the adjacent cranial architecture. A recent study demonstrated that NK cells are detectable among different bones, including the calvaria. Calvaria displayed the highest pro‐inflammatory signature compared with other bones [67]. Notably, calvarial marrow has been identified as a source of tumor‐associated neutrophils, which enhance T cell cytotoxicity, promote immune memory, and exhibit therapeutic potential in glioblastoma. Thus, NK cells in the calvaria may also interact with CNS‐resident immune cells, warranting further investigation [68].

### 4.2 Crosstalk Between NK Cells and CNS Resident Cells

The crosstalk between NK cells and other resident cell types in the CNS is worth exploring. Glial cells, such as astrocytes, microglia, oligodendrocytes, and neurons, are resident cells in brain parenchyma, where glial cells provide neuronal support and clear pathogens or dead neurons to maintain homeostasis [69]. Glial cells and neurons can produce inhibitory [e.g., transforming growth factor‐beta (TGF‐β) and interleukin‐10 (IL‐0)] or stimulatory [e.g., interleukin‐1 beta (IL‐1β), interleukin‐6 (IL‐6), TNF, M‐CSF, prostaglandin E2 (PGE2)] soluble factors to modulate NK cell activity in the CNS [70]. Besides, chemokines such as CX3CL1 secreted by neurons, CCL2 and CXCL10 released by astrocytes and microglia render NK cell recruitment into the inflamed brain [71, 72, 73, 74]. NK cells interact with these resident cells and reshape features to respond to the pathological CNS niche upon arrival. The ischemic neurons released CX3CL1 to attract NK cells to the infarct sites and ablated NK cell tolerance through lower expression of the NKG2A receptor ligand; thus, NK cells cause brain lesions and contribute to brain infarction by secreting perforin and IFN‐γ [75]. Similarly, astrocyte‐derived IL‐15 exacerbated ischemic brain injury by enhancing the activation and accumulation of NK cells and CD8^+^ T cells in injury sites [76]. However, astrocytes with human immunodeficiency virus (HIV) infection downregulated NKp44 ligand expression to escape from NK cell‐mediated cytotoxicity [77]. Microglia are CNS‐specialized macrophage‐like cells that respond quickly to pathological conditions and work as antigen‐presenting cells to prime reactive T cells. In EAE models, the interactions between microglia and NK cells determine the CNS inflammation magnitude and myelin‐reactive T helper 17 (Th17) cells, in which NK cells mitigate EAE severity by suppression of microglia and the following Th17 reaction augmentation [78]. Paradoxically, in the late stages of EAE models, NK cells approach the IL‐15‐producing neural stem cells and impede neurorepair capacity by elimination of the stem cells mediated by NKG2A ligand downregulation, suggesting that the removal or immune tolerance recovery of NK cells can ameliorate EAE [79]. These findings explicitly demonstrate that hyperactivated NK cells can act as pathogenic instigators to exacerbate central inflammation and tissue damage, suggesting that therapeutic strategies aimed at inhibiting or depleting specific destructive NK cell subsets could be highly beneficial in dampening autoimmune pathology. Thus, much remains to be done in understanding how the modulation of NK cells by CNS‐resident cells and the feedback by NK cells impact CNS homeostasis.

### 4.3 Immunosurveillance and Neuroregulation by NK Cells

The contribution to CNS homeostasis by NK cells can be classified into immunosurveillance and neuroregulation, depending on the physiological or pathological context. In EAE models, NK cells can reduce the induction of Th17 transcription factors by microglia, decrease RORα, RORγ, IRF4 transcripts, and STAT3 expression levels, thus in turn decreasing neuroinflammation [78]. NK cell depletion aborted the inhibitory effect of intravenous immunoglobulin (IVIG) on EAE models, while adoptive transfer of IgG‐treated NK cells suppressed EAE through induction of Tregs [80]. In a relapsing–remitting form of EAE models, IL‐2/IL‐2 mAb treatment attenuated CNS inflammation and neurological deficits, which is conferred by the expansion of CNS‐resident NK cells [81]. In experimental autoimmune uveitis (EAU) models, NK cells produce IFN‐γ to rescue the CXCR3 ligand production from DCs in IFN‐γ knockout mice and contribute to the IFN‐γ–IL‐27 self‐amplifying positive feedback to limit autoimmunity, suppress the growth of pathogenic Th17 cells, and promote the growth of T regulatory type 1‐like cells that produce IL‐10[82]. Though Natalizumab treatment reduces pathogenic T cells and alleviates EAE symptoms, it also significantly decreases NK cell frequency in the CNS. Natalizumab is linked to progressive multifocal leukoencephalopathy, often seen in immunocompromised individuals. Thus, the results suggested that the redundant depletion of NK cells may contribute to disturbance of CNS immune surveillance [83]. Beyond immunomodulation, NK cells also have a variety of functions that can be either neurotoxic or neuroprotective. The systemic depletion of NK1.1^+^ cells reduced anxiety‐like behavior and short‐term or long‐term memory abilities caused by inadequate release of IFN‐γ and acetylcholine, suggesting that ILC1/NK cells promote memory and vigilance behavior [65]. Besides, NK cells showed protective effects in Parkinson's disease (PD) patients due to the internalization and degradation of α‐synuclein (α‐syn) aggregates via the endosomal/lysosomal pathway, while depletion of NK cells in experimental PD models led to increased α‐syn aggregates and advanced progression [84]. Thus, these findings highlight the context‐specific roles of NK cells in different CNS disorders. Further studies are warranted to refine disease‐specific NK cell subsets and dissect how distinct NK cell subsets influence neurological outcomes.

In summary, NK cells in the CNS undergo continuous phenotypic reshaping driven by crosstalk with the neural stroma and evolving pathological states. Therefore, therapeutic success relies on mastering localized tissue signals within specialized reservoirs.

## 5 NK Cells in CNS Pathology

While homeostatic NK cells strictly guard the CNS borders, disrupted neurovascular barriers under pathological conditions allow these effectors to directly shape disease trajectories. The functional consequences of this infiltration vary significantly across different disease etiologies, spanning autoimmune neuroinflammation, neurodegenerative aggregates, and malignant tumors.

### 5.1 Autoimmune Disorders

#### 5.1.1 Multiple Sclerosis

Multiple sclerosis (MS) is an autoimmune disease that affects the CNS and locomotor system. It is mediated by abnormal inflammation and demyelination in the brain and spinal cord due to the overactivation of the immune system, leading to communication problems between the CNS and the rest of the body. Though the underlying pathogenesis remains poorly understood, MS is characterized by autoreactive T cell accumulation into the CNS. Many studies demonstrated that NK cells exert an immunoregulation function in MS by inhibiting overactive T cells through cytotoxicity capacity and regulatory cytokine release, while promoting NK cells’ contribution to MS remission [66, 85]. In line with these, some studies reported that MS susceptibility loci were also significantly enriched in genes relating to innate immune cells, including NK cells [86]. For instance, CD226, which is crucial for NK cell‐mediated elimination of activated T cells, showed lower expression in MS patients than healthy controls [87, 88, 89]. However, another study showed that NK cells were retained in the subventricular zone during brain inflammation and hindered the recovery postpeak phase by disturbing neurogenesis [79]. Thus, the contribution of NK cells to MS remains controversial.

In humans, the contribution of immature NK cells (CD16^dim^CD56^bright^) and cytotoxic NK cells (CD16^bright^CD56^dim^) to MS pathogenesis should be discussed separately. Since immature NK cells preferentially express higher levels of C‐C chemokine receptor 7 (CCR7), CD62L, CXCR3, and CXCR4 to respond to chemokines, it is no wonder that immature NK cells dominate in the CSF either in MS patients or healthy controls [12, 66, 90, 91]. Remarkably, MS patients showed a significant increase in the immature to cytotoxic NK cell ratio when compared with non‐MS or noninflammatory neurological disorders, and immature NK cells were negatively correlated to MS lesions by MRI, implicating that the accumulation of immature NK cells in the CSF of MS patients may limit CNS inflammation [91, 92]. Further evidence supports this, as successful MS therapy associates with the expansion of immature NK cells [93, 94, 95, 96]. Interferon β (IFN‐β) is widely used as a first‐line treatment for relapsed MS, as it can directly increase the expression of anti‐inflammatory factors and inhibit the production of proinflammatory cytokines. Clinical studies showed that IFN‐β treatment significantly increased the immature NK cell percentage in peripheral blood mononuclear cells and more in secondary lymphoid tissues, suggesting that immature NK cells expand and migrate into lymphoid tissues to interact with autoreactive T cells for inflammation control [93, 97]. Alemtuzumab is a humanized mAb with an indication for relapsed MS by depletion of CD52^+^ activated T and B cells and reconstruction. Recent studies showed that Alemtuzumab treatment expands immature NK cells, and the immature NK cell number influences the effector T cells, regulatory T cells, and B cells, suggesting the contribution of immature NK cells in immunity reconstitution triggered by Alemtuzumab [95, 98, 99]. Natalizumab is another mAb for relapsed MS by inhibiting transmigration of autoreactive T cells through blocking the α4 integrin. Immature NK cells are increased after 6 and 12 months of natalizumab treatment, indicating a possible immunoregulatory role of these NK cells [96]. Dimethyl fumarate (DMF) is approved as a first‐line treatment for MS due to the success of controlling relapsing MS and reducing new or enlarging MRI lesions [100]. Mechanistic studies showed that DMF exerts neuroprotective effects via the activation of Nrf2 and inhibition of NF‐κB [101, 102]. Similar to other MS immunotherapies, DMF treatment additionally increased immature NK cell number, while this was not observed in patients with poor responses to DMF. Besides, immature NK cells were inversely correlated to IFN‐γ and TNF‐α‐producing CD8^+^ T cells, indicating their essential roles in MS remission [94, 103]. Autologous hematopoietic stem cell transplantation has proven efficient in relapsing MS since the transplanted stem cells allow reconstitution of nonpathogenic immune system. Both immature and cytotoxic NK cells increased during the reconstitution, while immature NK cells replenished faster. Also, NK cells showed NKG2D‐dependent cytotoxicity to memory (CD45RO^+^CD45RA^−^) CD4^+^ T cells derived Th17 cells, which emphasized the importance of NK cells in limiting the autoreactive T cells after the hematopoietic stem cell transplantation treatment [104]. Taken together, these clinical studies suggest that immature NK cells are associated with positive outcomes in various immunotherapies for MS; however, further mechanistic studies are needed to clarify their precise role.

EAE has been utilized to dissect the role of immature NK cells in MS. The manipulation of NK cells in EAE models has proven their importance in inflammation control. Some studies showed that depletion of NK cells or inhibition of NK cell recruitment toward the CNS led to disease exacerbation, and expansion of NK cells with IL‐2 mAb complexes mitigated the disease. In these studies, NK cells restrict CNS inflammation by limiting autoreactive T cells, microglia, and macrophages [78, 105]. Thus, several studies demonstrated the positive roles of restoring NK cell activity in EAE models. Qa‐1, the mouse counterpart of human leukocyte antigen E (HLA‐E), is the ligand of CD94/NKG2A and is usually downregulated in EAE models, while the NKG2A blockade ablates the tolerance of pathogenic T cells or myeloid cells and significantly diminishes the EAE progression [106, 107]. Choline acetyltransferase (ChAT) can control the overactivated immune response; thus, the adoptive transfer of ChAT^+^ NK cells significantly reduced brain and spinal cord damage after EAE induction by disruption of the Qa‐1–NKG2A signaling and ablation of CCR2^+^Ly6C^hi^ monocytes [108, 109, 110]. IVIG shows protection in EAE, potentially by targeting Fc gamma receptors, which are highly expressed on NK cells. Hence, adoptive transfer of IgG‐treated NK cells suppresses EAE through induction of Tregs, while NK cell depletion abolished the therapeutic effects of IVIG [80]. TRAIL expression in astrocyte‐like cells can prime TRAIL‐DR5 signaling to induce T cell apoptosis. Though the process is impaired by T cells and microglia during CNS inflammation, it can be recovered by the IFN‐γ produced by meninges‐located NK cells [85].

Unlike immature NK cells, the contribution of cytotoxic NK cells is less discussed in MS due to the scarcity in the CNS context. A previous study reported increased percentages of cytotoxic NK cells with perforin expression in patients with progressive MS, suggesting a possible role in MS pathogenesis [111]. However, other studies demonstrated that the cytotoxic NK cells to IL17A^+^CD4^+^ T cells ratio and the cytotoxic NK cells to follicular Th cells (Tfh) cells ratio were significantly lower in MS patients with an exacerbated course, which is promising in distinguishing relapsing MS course and remitting course or healthy states [111, 112]. Previous studies showed that Epstein‐Barr virus (EBV) infection greatly increased the risk of subsequent MS, and EBNA1, an EBV protein, may trigger cross‐reactivity to glial cells and contribute to MS, while cytotoxic NK cells contribute more to the eradication of EBV infection than immature NK cells [113, 114]. Indeed, a more recent study demonstrated that NKG2C^+^ and NKG2D^+^ NK cell responses can efficiently control autoreactive B cells and T cells, while these responses are severely impaired in MS patients [115]. Thus, there remains inconsistency about how cytotoxic NK cells contribute to MS progression.

In accordance with these, some EAE studies showed the pathogenic rather than protective effects of NK cells. Systemic depletion of NK cells demonstrated a decrease in clinical pathology and disease frequency. Among these studies, IFN‐γ production by NK cells promotes the development of autoreactive Th1 cells and M1 macrophages, so the depletion of NK cells or neutralization of IFN‐γ delayed the onset of EAE [116, 117, 118]. Another study showed that IL‐15 produced by neural stem cells increased IFN‐γ‐expressing NK cells as well as NKG2D^+^ NK cells, while these NK cells suppress neural stem cells by direct cytotoxic effects [79]. However, these conflicting findings need further exploration, as the cytotoxic and immature NK cell subsets were not well defined in mice. CD27 was recently identified as a surface marker to distinguish cytotoxic and immature NK cell subsets in mice. A recent study found that CD27^−^ cytotoxic NK cells promoted DC maturation and aggravated disease after adoptive transfer in EAE models, whereas CD27^+^ immature NK cells inhibited CD4^+^ T cell proliferation and Th17 cell differentiation in vitro [119]. In contrast, another study reported that ozanimod treatment halted EAE progression and increased NKG2D expression of CD27^−^ NK cells rather than CD27^+^ NK cells in the CNS [120]. Thus, one NK cell subset may contribute to EAE through distinct mechanisms, necessitating further research into deeper stratification of NK cell subsets and interaction mechanisms with other immune cells.

Another question is the translational gap between EAE models and MS patients. Here, we take Natalizumab as an example. In EAE models, Natalizumab significantly decreases NK cell frequency in the CNS and the peripheral lymph nodes, as it can inhibit active immune cells. However, after Natalizumab treatment, peripheral NK cells increased 12 months later since the immune system of MS patients already underwent reconstitution, which is difficult to replicate in EAE models [83, 96]. Collectively, these results demonstrate the dual role of NK cells in MS pathology, where they may act as either disease agitators or regulators. Notably, the immature NK cell subset exhibits beneficial immunomodulatory effects in MS pathogenesis and could be harnessed as a therapeutic target.

#### 5.1.2 Neurotropic Viral Infection and Autoimmunity

As frontline effectors, NK cells provide a critical first‐line defense against neurotropic viruses to limit early replication. For example, herpes simplex virus type 1 antigens activate NK cells through toll‐like receptors (TLR) to prime infection control, while NK cell deficiencies cause susceptibility to severe, recurrent, or fatal infections [121, 122, 123]. Similarly, Zika virus exposure activates NK cells in the brain, upregulates the checkpoint receptor LILRB4, and promotes IFN‐γ and granzyme B accumulation in neonatal mice [124]. However, brain‐infiltrating NK cells can drive immunopathology in some cases. For instance, cytomegalovirus‐induced microglial CXCL9/CXCL10 chemokines recruit NK cells into the brain via CXCR3, where they fail to clear the virus and instead drive IFN‐γ‐dependent neurodevelopmental defects. This critical balance between antiviral defense and host immunopathology highlights the complex, dual‐natured role of NK cells, which becomes particularly vital when viral triggers lead to postinfectious autoimmunity.

Guillain–Barré syndrome (GBS) is the most common cause of acute flaccid paralysis due to peripheral nervous system damage resulting from the presence of autoreactive immune cells and autoantibodies. GBS histologically divides into acute inflammatory demyelinating polyradiculoneuropathy, characterized by T cell and macrophage infiltration with macrophage‐mediated demyelination, and acute motor axonal neuropathy, which is mediated by IgG antibodies targeting gangliosides without demyelination [125, 126]. GBS is strongly associated with antecedent infections such as *C. jejuni*, *Mycoplasma pneumoniae*, hepatitis E virus, Zika virus, and SARS‐CoV‐2, potentially triggering immune responses to autoantigens through molecular mimicry, as seen with the lipo‐oligosaccharides of *C. jejuni*, resembling gangliosides [127, 128, 129, 130, 131, 132, 133, 134, 135].

The activity of peripheral NK cells in GBS patients was functionally impaired and recovered to normal levels after plasma exchange therapy [136]. The NK cell deficiency may be caused by the presence of inhibitory cells or antibodies toward NK cells and exhaustion due to overactive T cells [137, 138, 139]. Besides, adoptive transfer of tolerogenic DCs ameliorated clinical symptoms of experimental autoimmune neuritis and increased numbers of NK and NKT cells [140]. Crucially, the role of NK cells in GBS is highly subset‐specific, with distinct subpopulations exerting diametrically opposed effects. Interestingly, in experimental autoimmune neuritis, which recapitulates GBS in rats, C‐X‐C chemokine receptor 5 (CXCR5) negative NK cells suppressed Tfh and showed the potential to control the humoral immune response, while CXCR5^+^ NK cells positively correlated with Tfh [141]. Consistently, a human study reported the positive correlations between GBS disability and CD56^bright^ NK cells and activated T cells in the CSF, while CD56^dim^ NK cells dominated after intravenous immunoglobulins or plasma exchange therapy, highlighting the distinct roles of NK cell subsets in the GBS pathogenesis [142]. These studies demonstrated that cytotoxic NK cells exert protective effects in GBS, while CD56^bright^ NK cells may exacerbate disease pathogenesis, which contrasts with observations in MS, suggesting disease‐specific immunomodulatory mechanisms in neuroinflammatory disorders. Consequently, rather than relying on uniform activation, optimal clinical outcomes in certain autoimmune contexts may heavily depend on targeted therapies that precisely inhibit these detrimental, inflammation‐promoting NK cell populations. Therefore, more research is required to determine the role of NK cell heterogeneity in the pathogenesis of GBS.

#### 5.1.3 Uveitis

Uveitis is an ocular inflammatory disease responsible for 10%–15% of global blindness and shares critical pathogenic mechanisms with MS, particularly through autoreactive T cell‐mediated inflammation. Our previous study showed the engagement of NK cells in restricting autoimmune uveitis pathogenicity. In IFN‐γ^−/−^ EAU models, IFN‐γ^+/+^ NK cells were recruited by DCs to the draining lymph nodes in a CXCR3‐dependent manner. This NK–DC interaction triggered IL‐27 production by DCs, which further amplified IFN‐γ secretion by NK cells, establishing a self‐reinforcing loop. Importantly, NK cell‐derived IFN‐γ and the subsequent DC‐derived IL‐27 suppressed pathogenic IL‐17 responses and promoted the generation of IL‐10‐producing type 1 regulatory T‐like cells, orchestrating an IFN‐γ–IL‐27–IL‐10 axis to control autoimmune inflammation [82]. Another study implicated CD8^bright^CD244^bright^ NK cells in HLA‐A29‐positive birdshot uveitis. CD8^bright^CD244^bright^ NK cells, a subset of cytotoxic NK cells with higher levels of IFN‐γ and TNF‐α, correlate with disease activity and decrease upon successful treatment with systemic immunomodulatory therapy in patients with birdshot uveitis [143]. These results reflect functional heterogeneity among NK subsets and context‐dependent mechanisms. Thus, further studies should refine NK cell diversity and spatial distribution to guide targeted therapies.

### 5.2 Neurodegenerative Diseases

Though neurodegenerative diseases are age‐related disorders featuring progressive neuronal loss, neuroinflammation is a crucial player in the process as well. According to new research, NK cells play a part in neurodegenerative illnesses such as AD, Parkinson's disease (PD), and amyotrophic lateral sclerosis (ALS).

#### 5.2.1 Alzheimer's Disease

AD is accompanied by cognitive decline and memory loss due to the presence of soluble Aβ plaques and hyperphosphorylated tau protein‐induced neurofibrillary tangles. AD can be categorized into two subtypes: early‐onset AD usually occurs before 65 and is featured by amyloid precursor protein (APP) or presenilin 1 or 2 (PSEN1/2) gene mutations, while late‐onset AD begins after 65 and is associated with apolipoprotein ε4 allele presence [144]. Single‐cell RNA sequencing showed the reduction of cytotoxic and adaptive NK cell subsets in AD patient peripheral blood, while a unique CX3CR1^+^ T‐bet^+^ NK cell subset increased with reduced NK cell cytotoxicity [145]. Though decreased cytotoxic function of NK cells in AD patients has been reported, some studies demonstrated that NK cells from AD patients showed an increased production of TNF‐α and IFN‐γ and higher cytotoxic capacity [146, 147, 148, 149]. Other studies reported NK cell cytotoxicity increased in patients with mild cognitive impairment but decreased in late‐stage AD patients, though it is unclear if NK cells contribute to or mitigate the AD severity [150]. In 3xTgAD models, NK cells in the brains are predominantly cytotoxic subsets with enhanced proinflammatory profiles such as *Gzmb*, *Ctsd*, *Ctsc*, and systemic depletion of NK cells reduced the proinflammatory property of microglia, drastically alleviated neuroinflammation, and improved cognitive function [151]. However, the administration of NK cells eliminated Aβ plaques, promoted a marked improvement in behavior in APP/PSEN1 mutant mice, and downregulated disease‐associated microglia‐related gene expression [152]. Hence, the NK cell subsets must be clearly defined before asserting a role for NK cells in the pathogenesis of AD. The effects of drugs on NK cells for AD treatment may also support the role of NK cells in AD pathology. Atorvastatin, a statin compound for cholesterol level control, can attenuate neural damage and improve cognitive function in rat models. When treated with atorvastatin, the expression of NK markers and IFN‐γ returned to normal levels, suggesting that atorvastatin decreases NK cells and prevents microglia activation by inhibiting IFN‐γ secretion [153]. SNK01 is a promising autologous NK cell therapy for AD patients due to its ability to eliminate activated T cells, degrade protein aggregates, and secrete anti‐inflammatory cytokines. The phase I study demonstrated that SNK01 was well tolerated and had beneficial effects on decreasing protein aggregate levels and neuroinflammatory biomarkers in the CSF [154]. Together, these results suggest an undervalued role of NK cells in AD pathology. Besides, further studies are needed to investigate if NK cell heterogeneity contributes to inconsistent results in AD research.

#### 5.2.2 Parkinson's Disease

PD is characterized by the loss of dopaminergic neurons in the substantia nigra and the accumulation of intracellular aggregates of α‐syn, which form Lewy bodies that can activate innate and adaptive immunity. The percentage of NK cells varies between blood and CNS in PD patients. Some studies showed a remarkable increase in NK cells in peripheral blood from PD patients, and the absolute number and cytotoxic activity of peripheral blood NK cells increased significantly in the early stage of PD [155, 156]. Moreover, a single‐cell RNA sequencing study reported a marked negative correlation between proportional decline of NK cells and PD severity, and NK subpopulations overexpressing X‐C motif chemokine ligand 2 (XCL2), a chemokine known to recruit X‐C motif chemokine receptor 1 (XCR1) positive cross‐presenting DCs into tumors, are involved in the occurrence and development of PD [157]. Similarly, recent studies revealed a positive association between NKG2D expression and PD patients with severe symptoms or a longer course, as well as lower NKG2A expression [158, 159]. On the contrary, another study reported an increase in less toxic NK cells (CD57^−^CD28^−^, CD57^+^CD28^−^) and a decrease in highly toxic NK cells (CD57^+^CD28^+^) in PD patients with prolonged duration and disease severity [160]. In the brain, NK cells are present in the substantia nigra, especially close to Lewy bodies of PD patients [84]. Moreover, the ratio of NK cells increased in the CSF of PD patients, especially the RAC1^+^ NK cells, indicating the potential of NK cells in neuroprotection and neurodevelopment [161]. However, the migration of NK cells from the peripheral blood to the brain remains elusive since the very late antigen‐4 (VLA‐4) expression of NK cells decreased in PD patients; thus, some compensatory pathways exist to partially offset the VLA‐4 deficiency [162]. In PD animal models, systemic depletion of NK cells resulted in exacerbated α‐syn pathology and motor deficits. Besides, in vitro studies showed that NK cells degrade α‐syn in TLR‐2‐ and TLR‐4‐dependent pathways, while α‐syn, in turn, represses NK cell cytotoxicity and IFN‐γ production, suggesting the potential of NK cells in PD control by targeting the abnormal proteins [84]. To date, PD can be alleviated but not cured. Besides, the medication for PD symptoms improvement may also affect NK cell activity since Levodopa increases serum IL‐15 levels in PD patients [163]. Interestingly, SNK01 has also been cleared for a phase 1/2a clinical trial in PD patients due to the expected ability to clear protein aggregates [164]. However, it remains unclear whether this adoptive transfer therapy influences the redistribution of immune cells. Overall, it is essential to clarify the contributions of different NK cell subsets to PD progression, as this may help explain the seemingly contradictory findings in some studies. What's more, it remains uncertain whether the increased activity of some NK cell subsets contributes to the progression of PD or is a consequence of unsuccessful attempts to counteract its progression.

#### 5.2.3 Amyotrophic Lateral Sclerosis

ALS is characterized by the progressive degeneration of motor neurons, which leads to muscle weakness and eventual paralysis. Though the cause of sporadic cases remains unclear, familial ALS is caused by mutations in various genes, including C9orf72, TAR DNA‐binding protein (TDP), superoxide dismutase 1 (SOD1), and fused in sarcoma (FUS) RNA‐binding protein [165]. Thus, animal models for ALS studies are developed based on these gene mutations, such as SOD1^G93A^, SOD1^G73R^, and TDP‐43^A315T^ models, which represent familial ALS. In humans, the peripheral NK cells exhibited either an increased or decreased trend in ALS patients in different studies [166, 167]. Further characterization of NK cell subtypes may reveal differences between ALS patients and healthy individuals. For example, cytotoxic NK cells with enhanced trafficking activity increased in ALS patients, suggesting NK cells are capable of migrating to inflammatory sites and dispatch damaged cells [168]. However, cytotoxic NK cells from ALS patients correlated with faster 50% progression, while immature NK cells associated with slower 50% progression, enhancing the prediction of ALS progression [169]. Similar findings were observed in sporadic ALS patients, where single‐cell RNA sequencing revealed that cytotoxic NK2 subsets with high expression of granzyme B and CD16, as well as NK4 subsets representing a transitional state to NK2, were significantly upregulated in ALS [170]. In SOD1^G93A^ mice, NK cells infiltrated the CNS and exhibited higher expression of granzyme B and CD69, a process mediated by CCL2. Notably, depleting NK cells with anti‐NK1.1 antibodies at an earlier stage significantly improved survival rates, associated with an increase in homeostatic neuroprotective microglia cells and Tregs [167]. Interestingly, another study showed a modest but not significant increase in survival after NK cell depletion in female SOD1^G93A^ mice [168]. Another study also showed that SOD1^G93A^ mice with TIR‐domain‐containing adapter‐inducing interferon‐β (TRIF) deletion experienced exacerbated disease development but showed reduced NK cell infiltration in the spinal cord [171]. The divergence may be attributed to treatment timing and NK cell heterogeneity. Thus, a more detailed characterization of NK cell subsets may be necessary to explain the seemingly contradictory results. To our knowledge, while the approved therapies for ALS (Riluzole, Edaravone, and AMX0035) have not been studied for their effects on NK cells, Tofacitinib has been shown to suppress IL‐15‐induced activation in both NK cell lines and primary NK cells from ALS patients. Therefore, a clinical trial study (NCT06689982) is set to initiate participant recruitment to evaluate the potential of tofacitinib in improving the disease course of ALS [172]. Overall, these data demonstrate a trend suggesting the involvement of cytotoxic NK cells in ALS and highlight the potential of targeting NK cells as a therapeutic approach for the disease.

### 5.3 Glioblastoma

Glioma is a heterogeneous group of tumors that can affect individuals of any age or gender, with approximately 310,000 new cases and 250,000 deaths in 2020 [173]. Glioblastoma (GBM), currently classified as an isocitrate dehydrogenase 1 wild‐type (IDH1^wt^) tumor of CNS WHO grade 4, represents the most common type of malignant brain tumor, accounting for approximately 50% of malignant cases in the glioma [174, 175, 176]. Despite the standard therapeutic regimen, the median survival and 5‐year survival rate of GBM remain low due to the tumor's resistance and recurrence, which are driven by factors such as the tumor microenvironment (TME), BBB, and cancer stem cells (CSCs) [177, 178]. Recent studies highlight the complex interaction between NK cells and GBM: while NK cells can target CSCs in GBM, their antitumor efficacy and tumor site infiltration can be hampered by the TME and BBB, respectively. Thus, it is important to understand NK cell plasticity within GBM to improve the efficacy of NK cell‐based immunotherapy.

#### 5.3.1 Tumor Microenvironment

The TME is mainly represented by microglia, astrocytes, macrophages, myeloid cells, blood vessels, and extracellular matrix, with miscellaneous immune cells [179]. While GBM cells theoretically represent ideal targets for NK cells due to their low MHC expression, NK cell antitumor activity is significantly suppressed within the GBM TME. For example, GBM has the tendency where the ratio of infiltrating cytotoxic and immature NK cells decreased with disease severity. The immature NK cells were enriched in the IDH1*^wt^* GBM, whereas cytotoxic NK cells were predominantly accumulated in the IDH1‐mutant (IDH1^mut^) GBM and brain metastases, a difference potentially attributable to CX3CL1 upregulation driven by the IDH1 R132H mutation [180, 181]. However, regardless of GBM subtype, NK cell efficacy is broadly impaired within the GBM TME through multiple mechanisms: the proportion of NK cells in GBM‐infiltrated immune cells remains low, accounting for less than 1% [182]. Besides, NK cells infiltrated in GBM harbor insufficient cytotoxic phenotypes such as decreased expression of NKG2D, NKp30, DNAM1, and CD317, which is partially mediated by TGF‐β secreted by cancer cells and microglia, macrophages, and MDSCs in the TME [183, 184]. Moreover, NK cells may lose targets or ablate cytotoxicity due to the ligand expression by GBM cells such as MHC‐I, PD‐L1, and CD73 [185, 186]. Despite these challenges, NK cells can reciprocally influence the TME through crosstalk with other cells. For example, NK‐cell‐derived IFN‐γ is critical for the accumulation of blood monocyte‐derived CD169^+^ macrophages in GBM, which produce pro‐inflammatory chemokines, CCL5 and CXCL10, creating a self‐reinforcing antitumor niche by recruiting additional NK cells [187]. Therapeutic strategies to overcome NK cell suppression show promise, as evidenced by the adoptive transfer of engineered NK cells, which countered TIGIT and CD73 signaling, improved NK cell function in intracranial patient‐derived xenograft models of GBM, and resulted in complete tumor eradication [188].

In addition, the unique metabolic and physical characteristics of TME contribute to immune evasion and drug tolerance in GBM. Hypoxic stress in the TME also contributes to the impaired NK cell phenotypes. Hypoxia‐inducible factor (HIF) promotes cell survival in the TME but generates new immunosuppressive features of immune cells [189]. Additionally, HIF‐1α‐deficient NK cells failed to infiltrate hypoxic zones, resulting in decreased sVEGFR1 levels, which led to poor angiogenesis and reduced tumor volume [190]. Thus, the inhibition of HIF‐1α promotes NK cell activation abilities, enhances the NK cell response to IL‐18, and unleashes the antitumor activities on NK cells [191]. A recent study demonstrated that preactivation of NK cells with cytokines (IL‐15, 4‐1BBL, and IL‐21) can confer resistance to hypoxic conditions, highlighting the therapeutic potential of engineering adoptively transferred NK cells to express intrinsic cytokines for enhancing immunotherapy efficacy [192]. Lactate is produced by cancer cells in aerobic glycolysis and impairs NK cell function and survival to cause immune evasion [193]. Lactate induced NK cell apoptosis through intracellular pH decrease and mitochondrial dysfunction, thus contributing to colorectal cancer liver metastases by the depletion of liver‐resident NK cells [194]. NK cells armed with autonomous IL‐21 secretion also showed metabolic fitness to glycolysis by enhanced oxidative phosphorylation and ATP production, supporting the long‐term persistence and antitumor activity in GBM [195]. The mechanical properties of TME also hinder NK cell activity. Stiffer substrates enhanced NK cell cytotoxicity with higher levels of IFN‐γ, granzyme A and B, and FasL, while GBM tumors were softer than the brain parenchyma, and with an increase in glioma grades, they tended to become even softer [196, 197, 198, 199]. Moreover, GBM tumor cells can alter their metabolism in response to their local mechanical properties, while tumor edges and adjacent tissues are softer than the tumor core and shift into glycolysis, inducing impaired NK cell energy metabolism through TME acidification [194, 199].

#### 5.3.2 Blood–Brain Barrier

Another major limitation to effective therapy is the BBB, which impedes the entry of most targeted drugs administered systemically to the lesion sites. Currently, Temozolomide (TMZ) and nitrosoureas are approved first‐line and second‐line drugs for GBM treatment, with a high efficacy for crossing the BBB of 15%–30%, the systemic toxicity limits the dosage for drug delivery. Multiple strategies are being developed to overcome or circumvent the BBB, such as improving drug penetration or BBB disruption [200]. For instance, TMZ plus liposomal doxorubicin was tested in recurrent GBM and showed promising results with a 19% overall response rate [201]. Significant mitigation was also observed in rat GBM models with adoptive transfer of HSP70/IL‐2‐treated NK cells, while systemic injection confirmed the migration of these NK cells across the BBB for tumor cell elimination [202]. Transient BBB disruption using ultrasound appears a promising approach for drug delivery. Compared with TMZ alone, combined focused ultrasound treatment increased the TMZ CSF/plasma ratio from 22.7% to 38.6% in GBM rat models [203]. In the xenograft human epidermal growth factor receptor 2 (HER2) positive breast cancer brain metastasis model, the combination treatment with targeted NK‐92 cells and MRI‐guided focused ultrasound treatment improved survival compared with either treatment alone, resulting in 50% long‐term survival of subjects [204]. Recently, a first clinical trial (NCT02253212) also confirmed the good tolerance of ultrasound treatment combined with carboplatin, with increased effectiveness and prolonged median overall survival [205]. In addition, the tertiary lymphatic structures (TLS) have been found in proximity to brain tumors, which are alternative sites for T cell priming and may initiate immune responses to GBM bypassing the BBB and warrant further studies [206]. Another study reported TLS formation in GBM TME induced by intracranial administration of TLR agonists and glioma antigens and subsequent improved prognosis in glioma‐bearing mice. Though TLSs do not contain NK cells, further analysis in clinical data demonstrated that TLS^high^ clusters harbored augmented antitumor cells including NK cells, CD4^+^ T cells, CD8^+^ T cells, and immunosuppressive cells like M2 macrophages, Tregs, and MDSCs in the TME, indicating TLS clusters are characterized by high immune infiltration rather than an ‘immune‐desert’ [207, 208]. In summary, these studies support the potential of manipulation of BBB permeability to spur immune responses, which has broader implications for brain malignancies.

#### 5.3.3 Cancer Stem Cells

CSCs are dormant stem cells concealed inside tumors, characterized by self‐renewal and multi‐ or pluripotent abilities, and are regarded as the primary contributors to tumor recurrence, metastasis, and poor response [209]. Some studies suggested that NK cells may preferentially recognize and lyse CSCs in various types of cancer, including GBM and melanoma [210, 211]. CSCs trigger NK cell cytotoxicity due to the increased expression of different specific activating ligands and the decline or lack of expression of MHC‐I on CSCs [210, 212, 213]. For example, CSCs from GBM patients were highly sensitive to IL‐2/IL‐15‐activated NK cells because they expressed high levels of PVR and Nectin‐2, the ligands of DNAM‐1‐activating NK receptors [210]. Though CSCs are susceptible to NK cells, they may evade NK cell surveillance by forming a CSC niche, inducing NK cell ablation, and adjusting resistance. First, CSCs sustain stemness and insulates surveillance by forming a niche that includes immune cells, stroma cells, and cytokines [214]. NK cell cytotoxicity is impaired by MDSCs with low expression of NKG2D and IFN‐γ secretion [215]. TGF‐β, IL‐6, and IL‐10 produced by Tregs and DCs promote NK cell inhibition [216, 217]. Prostaglandin E2, indoleamine 2,3‐dioxygenase, and TGF‐β secreted by cancer‐associated fibroblasts reduce the perforin and granzyme B expression on NK cells [218]. A recent study reported that in a patient‐derived xenograft model of GBM, NK cell dysfunction induced by CSCs was completely prevented by direct blockade of αv integrin or TGF‐β, as well as through gene editing of the TGF‐β receptor 2 on NK cells, resulting in effective tumor control [219]. Furthermore, CSCs express checkpoint ligands such as PD‐L1 and disrupt NKG2D expression to induce NK cell exhaustion [220, 221]. For example, GBM CSCs generated extracellular vesicles with PD‐L1 expression, thus blocking T cell activation and proliferation [222]. CSCs from GBM express high levels of enhancer of zeste homolog 2 (EZH2)‐92aa, which directly represses MICA/B transcription by binding the promoters and indirectly suppresses ULBP by stabilizing EZH2 to induce NK cell resistance [223]. miR‐20a, miR‐93 and miR‐106b downregulate the expression of NKG2D ligands in GBM CSC spheres and protect CSCs from NK cell‐mediated lysis [224].

In conclusion, NK cells exhibit highly polarized, context‐dependent roles across CNS disorders, acting either as pathogenic instigators of neuroinflammation or as suppressed tumor effectors. Therefore, effective clinical translation requires subset‐specific interventions to either strategically inhibit destructive NK cells in autoimmune settings or deploy advanced multispecific engagers against glioblastoma.

## 6 Therapeutic Strategies for CNS Diseases by Targeting NK Cells

Given the contrasting roles of NK cells across CNS pathologies, clinical translation requires disease‐specific therapeutic bifurcations. In glioblastoma (GBM), interventions must amplify NK cell cytotoxicity to dismantle malignant tissues, whereas in neuroautoimmune disorders, strategies must downregulate overactivated pathogenic NK subsets to mitigate neuroinflammation. Accordingly, current pipelines utilize a distinct spectrum of molecular modulators, multispecific engagers, and advanced adoptive cell engineering to target these diverse neurological lesions.

### 6.1 Antibody, Cytokine, and Engager‐Based Modulations

**FIGURE 3:**
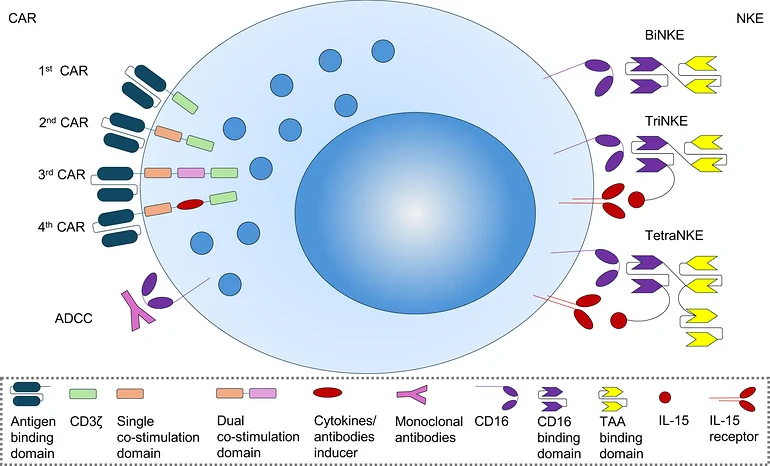
NK cell‐based therapies for neurological diseases. ADCC relies on the binding between monoclonal antibodies and CD16 to activate NK cells and target aberrant cells. Besides, more research focuses on CAR‐NK and NK cell engagers to enhance NK cell targeting and activation. The BiNKE binds to CD16 to prime ADCC and bridge NK cells to aberrant cells by targeting the antigen. TriNKE additionally introduces an IL‐15 element to promote NK cell expansion. TriNKE engages more antigens or activation receptors to activate NK cells. CAR‐NK changed from the 1st CAR, which was dependent on CD3ζ, to the 2nd and 3rd CAR, which included one or multiple co‐stimulatory domains, to the 4th CAR, which endowed NK cells with autocrine cytokines or soluble antibodies. ADCC, antibody‐dependent cellular cytotoxicity; BiNKE, bi‐specific NK cell engager; CAR, chimeric antigen receptor; CD3ζ, cluster of differentiation 3 zeta chain; IL‐15, interleukin‐15; IL‐15R, interleukin‐15 receptor; NKE, NK cell engager; NK, natural killer; TAA, tumor‐associated antigen; TetraNKE, tetra‐specific NK cell engager; TriNKE, tri‐specific NK cell engager.

As a powerful off‐the‐shelf alternative to cell transplantation, the development of NK cell engagers (NKE) can be particularly promising, as they enhance NK cell activation and recruitment to the TME by immunological synapse formation through linking tumor cell antigens and NK cell surface molecules (Figure 3) [225]. Multispecific NKEs are designed to target CD16 and tumor antigens to prime ADCC of NK cells, which showed preliminary safety and efficacy in clinical trials such as refractory relapsed lymphomas [226]. Other strategies involve bridging tumor cell antigens to NK cell surface markers, including CD16, NKG2D, NKp46, NKp30, IL‐2R, and IL‐15R, to enhance NK cell activation, promote NK cell expansion, and confer greater avidity simultaneously [227, 228]. A novel multispecific NKE targets CXCL10 and IL‐15R, sustaining NK cell activation by elevating local CXCL10 and IL‐15 levels, which promotes NK cell homing to the tumor microenvironment and enhances anti‐GBM responses, suggesting the potential of NKEs in overcoming TME in GBM [229]. In conclusion, NKEs demonstrate strong potency by modulating innate activation receptors expressed on NK cells, thereby improving their efficacy in combating the tumor.

### 6.2 Adoptive Cell and CAR‐NK Immunotherapy

Beyond molecular modulations, adoptive cell therapy has emerged as a frontline pillar in the translational landscape. In the immunosuppressive context of GBM, the most common strategies include the adoptive transfer of transgenic NK cells, some of which may carry modifications of cytokine secretions, helping to counteract the inert microenvironment after NK cell administration [230]. For instance, engineered NK cells with IL‐21 locoregionally persistent secretion show long‐term antitumor activity and safety, as IL‐21 drives epigenetic reprogramming of NK cells through C/EBP transcription factors and enhances anti‐GBM efficacy [195].

Adoptive cell therapy using CAR immunotherapy has made remarkable advancements since seven CAR‐T cell therapies have been approved by the US Food and Drug Administration for hematological malignancies [231]. However, CAR‐T cell therapy in solid tumors lags significantly behind due to the low efficacy and safety caused by limited tumor‐specific antigen selection, inefficient infiltration to tumor sites, cytokine release syndrome (CRS), and neurotoxicity [232]. Alternatively, CAR‐NK cell therapy addresses the unmet needs in CAR‐T cell therapy with several advances. First, NK cells act in an HLA‐unrestricted manner and rarely induce graft‐versus‐host disease, increasing clinical trials utilizing NK‐92 cell lines or primary NK cells from allogeneic donors for adoptive transfer therapy [7, 233, 234, 235]. Second, CAR‐NK cell therapy has multiple tumor recognition mechanisms due to the innate MHC‐ and KIR‐directed cytotoxicity and ADCC, which are CAR‐independent. Third, rare CRS or neurotoxicity events were reported in CAR‐NK cell therapy with no significant release of pro‐inflammatory cytokines such as IL‐1β, IL‐6, and TNF‐α [7, 236]. Thus, CAR‐NK cell therapy is currently being actively explored in preclinical and clinical studies in hematological malignancies and solid tumors such as GBM (Table 2). However, CAR‐NK cell therapy shares similar CAR constructs with CAR‐T cell therapy, from the first‐generation CAR, which was dependent on the CD3 zeta chain (CD3ζ), to the second‐ and third‐generation CAR, which included one or multiple co‐stimulatory domains (Figure 3) [237]. Notably, the fourth‐generation CAR adopted a completely new strategy that endows the CAR‐NK cells to adjust or modify the microenvironment by introducing autocrine cytokines or soluble antibodies [7, 237, 238]. For example, one multifunctional CAR‐NK cell therapy targeting GD2 and NKG2D ligands with anti‐CD73 antibodies locally released in the TME showed long‐term retention and effective antitumor activity in GBM xenograft models through overcoming the GBM heterogeneity and reprogramming the TME [239]. Another CAR‐NK cell therapy based on the NK‐92/5.28.z cells targeting the HER2 antigen showed potent tumor control in HER2^+^ GBM xenograft models and orthotopic models. Thus, a phase I trial, CAR2BRAIN (NCT03383978), is ongoing to evaluate the safety, tolerability, and antitumor activity of NK‐92/5.28.z in patients with HER2^+^ GBM [240, 241].

**TABLE 2:** Clinical trials referring to NK cell‐based therapies for neuroinflammation and glioblastoma.

Disease	Agent	Mechanism	Regimen	Study state	Identifier	References
MS	Allogeneic g‐NK cells	Engineered NK cells targeting autoreactive T and B cells	Administered in combination with IL‐2 and ocrelizumab	Phase 1b	NCT06677710	[242]
AD	Autologous NK cells	Engineered NK cells targeting activated T cells	Infusion	Phase 1/2a	NCT06189963	[154]
GBM	HER2 CAR‐NK‐92 cells	Engineered NK cells targeting HER2^+^ tumor cells	Intracranial injection in combination with intravenous Ezabenlimab	Phase 1	NCT03383978	[240]
GBM	Anti‐MUC1 CAR‐NK cells	Engineered NK cells targeting MUC1^+^ tumor cells	Intracranial injection	Phase 1	NCT02839954	[243]
GBM	Allogeneic NK cells	Haploidentical NK cells expanded with rhIL‐15	Intra‐CSF injection	Phase 1	NCT05108012	[244]
GBM	Allogeneic NK cells	Active allogeneic NK cells	Intrathecal injection	Phase 2	NCT06687681	[245]
GBM	Allogeneic NK cells	Haploidentical donor NK cells	Infusion	Phase 2	NCT02100891	[246]
GBM	Autologous NK cells	Ex vivo expanded NK cells	Infusion	Phase 1	NCT00909558	[247]
GBM	Autologous NK cells	Ex vivo expanded NK cells ± rhIL‐15	Infusion	Phase 1	NCT01875601	[248]
GBM	Autologous NK cells	*Ex vivo* expanded NK cells	Intratumoral injection	Phase 1	NCT04254419	[249]
GBM	Cord blood‐derived expanded allogeneic NK cells	Engineered NK cells with TGF‐βR2 and NR3C1 deletion	Intratumoral injection	Phase 1	NCT04991870	[250]

Abbreviations: AD, Alzheimer's disease; CAR, chimeric antigen receptor; CSF, cerebrospinal fluid; GBM, glioblastoma; HER2, human epidermal growth factor receptor 2; IL, interleukin; MS, multiple sclerosis; MUC1, mucin 1; NK, natural killer; NR3C1, nuclear receptor subfamily 3 group C member 1; rhIL‐15, recombinant human interleukin‐15; TGF‐βR2, transforming growth factor‐beta receptor type 2.

Beyond oncology, adoptive cell therapy is breaking ground in nonneoplastic neurological contexts. For example, SNK01, an autologous nongenetically modified NK cell, has been administered intravenously for a clinical study on AD due to the potential of internalization and degradation of Aβ and α‐syn aggregates and secretion of IL‐10 and TGF‐β [154]. Furthermore, clinical trials based on CAR‐NK cell therapy may also extend indications to autoimmune diseases, as growing evidence indicates that special NK cell subsets may target autoreactive T cells and B cells (Table 2). NKG2C^+^ NK cells can eliminate autoreactive GlialCAM_370–389_‐specific cells caused by EBV infection through the NKG2C–HLA‐E axis, suggesting their potential for the prevention and treatment of EBV‐induced MS [115]. g‐NK cells lack NKG2A but have elevated NKG2C expression, which primes the NKG2C‐HLA‐E axis to target autoreactive cells, and are being investigated in combination with ocrelizumab and IL‐2 in a phase 1b study for patients with refractory progressive MS (NCT06677710) [242]. Another CAR‐NK cell therapy targeting the CD19 antigen is under investigation in a phase 1 study in adults with systemic lupus erythematosus for evaluation of safety, tolerability, pharmacokinetics, immunogenicity, and efficacy (NCT06518668) [251]. However, CAR‐NK cell therapy is still a developing field that needs more research to confirm safety and efficacy in treating autoimmune diseases and neuroinflammation. Notably, lessons from Breyanzi, formulated in a 1:1 CD4/CD8 CAR‐T cell ratio with reduced CRS, provide valuable insights for advancing CAR‐NK cell therapies. Given NK‐cell heterogeneity, selectively expanding or engineering specific NK subsets may optimize the safety and efficacy of NK cell‐based immunotherapy in neurological disorders [252, 253].

## 7 Conclusion and Prospects

NK cells have emerged as pivotal regulators of immune homeostasis within the CNS, functioning beyond their traditional role as innate cytotoxic lymphocytes. As highlighted throughout this review, NK cell biology is characterized by remarkable phenotypic and functional heterogeneity, with distinct subsets exhibiting diverse activation states, migratory capacities, and effector functions. Their recruitment, activation, and adaptation within the CNS are tightly regulated by local cytokines, chemokines, resident cells, and the unique immune‐privileged environment, enabling NK cells to participate in immune surveillance while maintaining tissue integrity. The coordinated regulation of activating and inhibitory signaling pathways, together with tissue‐specific environmental cues, underlies the context‐dependent functions of NK cells in CNS homeostasis and disease. Increasing evidence indicates that NK cell plasticity and tissue‐specific adaptation are fundamental features that determine their protective or pathogenic functions across neurological diseases.

NK cells play an immunosurveillance role in different CNS diseases, capable of eliminating both autoreactive immune cells and tumor cells, yet their clinical application faces limitations due to incomplete knowledge of how specific subsets function within the CNS. To bridge this gap, future research should prioritize high‐resolution spatial mapping of NK cell dynamics across neurological regions, coupled with single‐cell profiling to define CNS‐adapted subsets beyond conventional classifications. Deciphering these spatiotemporal dynamics is essential to uncover how localized chemokine gradients and cellular niches sculpt distinct NK cell phenotypes. By moving beyond rigid surface‐marker classifications, these multi‐omic approaches will reveal tissue‐specific transcriptional networks and metabolic adaptations, providing a vital mechanistic framework to determine whether an infiltrating subset promotes tissue repair or drives secondary neuropathology.

For neuroinflammation or neurodegeneration, therapeutic development should focus on selective modulation of pathogenic NK subsets while preserving protective immunity, requiring careful consideration of CNS‐specific immune regulation. Global immunosuppression or indiscriminate NK cell activation often leads to off‐target inflammatory cascades or compromised host defense. Consequently, future translational pipelines must leverage precise molecular axes, such as the HLA‐E/NKG2C pathway or regional homing receptors, to selectively silence or deplete specific auto‐reactive NK cells responsible for axonal damage and demyelination. Transitioning to such subset‐specific interventions represents a critical prerequisite for safely steering the central immune landscape without disrupting baseline neurological homeostasis.

For GBM, emerging strategies in immune engager platforms and adoptive NK therapies must account for the CNS's immune‐privileged nature, particularly the BBB and immunosuppressive niche. Parallel advances can integrate TME‐responsive systems such as hypoxia or pH‐sensing CARs to enhance NK cell resistance in the inert GBM niche. By combining mechanistic insights from neuroinflammation and neuro‐oncology, we can advance precision NK cell therapies that maintain CNS homeostasis while effectively targeting disease—a crucial step toward developing clinically viable treatments that harness NK cells’ dual immunoregulatory functions. These bioengineered logic gates ensure that cytolytic activities are strictly restricted to the malignant core, mitigating peripheral toxicities and off‐target neuroinflammation. Ultimately, bridging the boundaries between neuro‐oncology and neuroimmunology will facilitate the development of next‐generation, bifunctional NK cell therapies capable of eradicating intracranial tumors while respecting the fragile microenvironment of the central nervous system.

Overall, recent advances in single‐cell RNA sequencing, spatial transcriptomics, and multifunctional bioengineered NK‐cell platforms integrating immune‐checkpoint modulation, cytokine support, and metabolic optimization are rapidly transforming our understanding of NK‐cell biology in the CNS and providing unprecedented opportunities for precision immunotherapy [254]. However, successful clinical translation will require integrating insights into NK cell biology, activation mechanisms, tissue‐specific dynamics, and disease‐associated microenvironments to accurately identify and therapeutically target functionally distinct NK cell subsets. Future therapeutic strategies should therefore move beyond indiscriminate NK cell activation or depletion toward precise modulation of disease‐associated NK cell populations tailored to distinct CNS pathologies. Ultimately, continued integration of neuroimmunology, neuro‐oncology, and bioengineering will accelerate the development of next‐generation NK cell‐based therapies that restore immune homeostasis, overcome the immunosuppressive CNS microenvironment, and provide safer and more durable therapeutic benefits for patients with CNS diseases.

## Author Contributions

K.D. and W.P.C. contributed to the conception or design of the work. K.D. wrote the original draft of the manuscript. W.P.C. critically revised the manuscript. Both authors read and approved the content of the manuscript before final submission.

## Funding

This work was supported by the National Natural Science Foundation of China (32370959), Hong Kong Baptist University (167664), and the Innovation and Technology Fund of Hong Kong (ITS/057/23MS).

## Ethics Statement

The authors have nothing to report.

## Conflicts of Interest

The authors declare no conflicts of interest.

## Data Availability

The authors have nothing to report.
